# Phenology‐informed decline risk of estuarine fishes and their prey suggests potential for future trophic mismatches

**DOI:** 10.1002/eap.70130

**Published:** 2025-11-10

**Authors:** Robert J. Fournier, Tyler C. Marino, Stephanie M. Carlson, Albert Ruhí

**Affiliations:** ^1^ Department of Environmental Science, Policy, and Management University of California Berkeley Berkeley California USA

**Keywords:** estuaries, food webs, phenology, population viability analysis, time series analysis

## Abstract

Conservation scientists have long used population viability analysis (PVA) on species count data to quantify critical decline risk, thereby informing conservation actions. These assessments typically focus on a single species rather than assemblages and assume that risk is consistent within a given life stage (e.g., across the different seasons or months of a year). However, assessing risk at overly broad temporal or spatial scales may obscure diverging population declines between predators and prey, potentially disrupting biotic interactions. In this study, we used time‐series‐based PVA for age‐0 forage fishes and their potential zooplankton prey for each month of the year in the San Francisco Estuary, over 1995–2023 (*N* = 175 time series). The PVA were parameterized using Multivariate Autoregressive (MAR) models that estimate long‐term population trends and variability (i.e., process error) for each population. We found widespread negative population trends across fish species (56.8%) and observed that critical decline risk is often higher in months when species peak in abundance compared to “shoulder” months. Although current decline risk is somewhat balanced between predators and their prey (mean 23.7% for fish and 21.1% for zooplankton), our time‐series models indicate trophic levels are poised to diverge over the next 10 years, with fish generally accumulating risk faster than their prey. Additionally, zooplankton showed 11.2% higher uncertainty about their near‐term critical decline risk relative to fish. These observations suggest strong, previously unreported potential for future trophic mismatches. Our results underscore the need to assess risk over finer temporal scales within and across trophic levels to better understand vulnerability, and thus inform conservation of imperiled species. Our approach is transferable and highlights the benefits of time‐series‐based PVA to understand risk of food‐web collapse in the face of climate‐induced phenological shifts.

## INTRODUCTION

Global climate change challenges current efforts to conserve and manage biodiversity (IPBES, 2019). Severe population declines and local extirpation can drive permanent shifts in community composition and destabilize whole food webs (O'Gorman & Emmerson, 2009; Seifert et al., 2015). Though conservation actions attempting to bolster population recovery are widespread geographically across both animal and plant systems (Havens et al., 2014; Mawdsley et al., 2009; Swaisgood, 2007), quantifying extinction risk in species with complex life cycles remains challenging (e.g., Daly et al., 2021; Sánchez‐Hernández et al., 2019). For instance, determining the effects of a given stressor (e.g., temperature, salinity) on individual survival or performance must account for the differential sensitivities across life stages (Komoroske et al., 2014). Additionally, species phenology (e.g., the timing of migration, breeding, or niche shifts) can cause the strength of important biotic interactions to vary through time and across space (Werner & Gilliam, 1984). Thus, accounting for community‐level dynamics, including how predators and prey coexist in space and time, is essential for improving conservation outcomes of at‐risk species.

Population viability analysis (PVA) is a widely used tool to assess threats to species persistence, forecast population trends, and guide recovery (Akçakaya & Sjögren‐Gulve, 2000). Historically, these models have taken many forms—from demographic assessments to minimum viable population size estimates to mechanistic, covariate‐driven simulations (Gerber & González‐Suárez, 2010). PVA allows conservation practitioners to assess population performance by estimating the cumulative probability of exceeding a critical decline threshold, often termed “quasi‐extinction” (Fagan & Holmes, 2006). However, these models generally have strict data requirements and are difficult to effectively parameterize without a robust understanding of demographic and environmental processes (Chaudhary & Oli, 2020). To circumvent this shortcoming, statistical methods were developed that estimate the convergent properties of stochastic systems and allow for quasi‐extinction forecasting even when the underlying mechanistic processes are poorly understood (Holmes et al., 2007). Such models use repeated sampling of a population (i.e., a time series of count data) to assess inherent growth rates and process‐driven population variability. In addition to applications estimating quasi‐extinction probabilities under different scenarios (e.g., Ruhi et al., 2018; Ruhí et al., 2016), these methods can also infer metapopulation spatial structure and associated risk (e.g., Holmes & Semmens, 2004; Ward et al., 2010). Given the increasing availability of long‐term biomonitoring datasets, understanding the potential and limitations of statistical approaches to species extinction forecasting is an important endeavor of conservation science.

Despite advances in time series‐based PVA, current approaches primarily use annual data (Hampton et al., 2013; Holmes et al., 2014). As such, species with sub‐yearly phenological patterns (e.g., hatching dynamics, age‐0 migrations; Bogner et al., 2016) might display temporal patterns of critical decline risk not captured by a coarser scale approach. Moreover, while it is possible to examine species interactions using multivariate time series models (e.g., Hampton et al., 2006; Peterson et al., 2017), estimation of interaction strengths often conflicts with estimation of PVA parameters (i.e., intrinsic growth rate or “lambda,” Holmes et al., 2014). However, food‐web dynamics that might be critical to population performance can be assessed indirectly—by comparing fine‐scale patterns of risk of a predator versus that of its prey. While statistically challenging, it is ecologically important to consider decline risk within the context of community‐level interactions, especially in environments where population persistence may be influenced by climate‐induced changes in species phenology.

Estuaries are dynamic ecosystems with levels of biological productivity comparable to tropical rainforests and coral reefs (Cai, 2011). Estuarine systems provide high socioeconomic value, facilitate important ecosystem services and govern many nearshore physical and biological processes (Barbier et al., 2011; Robins et al., 2016). For many taxa, estuaries often represent important nursery grounds (Beck et al., 2001; Colombano et al., 2020), refuge habitats (Simenstad et al., 1982), and migration corridors (Koeller et al., 2009; Otero et al., 2014). However, the transitional nature of estuaries makes them highly vulnerable to environmental change, as degradation to both the marine and freshwater bookends has the potential to disrupt estuarine communities (Gillanders et al., 2011; Lauchlan & Nagelkerken, 2020). Indeed, many estuarine systems globally are experiencing climate‐induced shifts in temperature and salinity regimes that can strongly impact population dynamics (Ghalambor et al., 2021; Langan et al., 2021; Scanes et al., 2020). As estuaries often act as temporary or transitional habitats for key life stages, many taxa have developed population cycles that maintain historical synchrony between interacting species (Marques et al., 2006). However, warming and salinization appear to be altering phenological patterns in estuarine food webs—with the potential to disrupt historically synchronous population cycles between predators and prey (Asch et al., 2019; Chevillot et al., 2017; Fournier et al., 2024). This is especially important for juvenile fishes, as global change drivers that disrupt community compositions could lead to recruitment failures that erode the nursery function of estuarine ecosystems (Colombano et al., 2022).

The San Francisco Estuary is one of the largest and most ecologically significant estuaries in North America, draining approximately 40% of California's fresh waters (Cloern & Jassby, 2012). The estuary spans a wide salinity gradient from the Pacific Ocean to the confluence of the Sacramento and San Joaquin rivers and is highly affected by both hydroclimatic variability and large‐scale water diversions for agricultural and municipal use (Reis et al., 2019). In the past decade, the estuary has experienced steadily increasing water temperatures (Bashevkin et al., 2022), and long‐term droughts have decreased freshwater inputs into the Delta, resulting in increased salinity levels in the upper estuary (Barros et al., 2024). Additionally, invasions of Asian clams (*Potamocorbula amurensis* and *Corbicula fluminea*) in the late 1980s dramatically eroded planktonic populations (Kimmerer et al., 1994), leading to dietary shifts of planktivores (Feyrer et al., 2003). These and other environmental changes have resulted in large‐scale collapses of pelagic fish populations throughout the estuary (Cloern & Jassby, 2012; Quiñones & Moyle, 2014). Often referred to as the “pelagic organism decline (POD),” forage fish populations have shown precipitous drops even during periods of relatively moderate abiotic stress (Sommer et al., 2007). Though the mechanistic causes of this decline remain poorly understood, recruitment failure, increased mortality, habitat degradation, and limited food availability have been identified as main drivers—especially for juvenile forage fish (Feyrer et al., 2007; Mac Nally et al., 2010; Sommer et al., 2007). These trends underscore the need to better understand the dynamics of forage fishes in the first year of their life (i.e., age‐0), as well as of their food sources.

Here, we sought to assess spatial and temporal patterns of critical decline risk of fish, and their suite of potential prey in the San Francisco Estuary. As age‐0 estuarine fishes display seasonally varying abundance patterns that might coincide with periods of population vulnerability (i.e., higher quasi‐extinction risk), we sought to examine critical decline risk at sub‐annual scales. To that end, we used long‐term monitoring data to conduct time‐series‐based PVA for each month of the year for age‐0 forage fishes and zooplankton taxa across different regions of the San Francisco Estuary spanning a broad environmental gradient. We hypothesized that (1) long‐term population trends and variability around those trends would vary across fish species, creating ample variation in the probability of them crossing critical decline thresholds (hereafter, *critical decline risk*); (2) critical decline risk in a given species would also vary across the year, with months that historically concentrated high abundance of age‐0 being relatively safer than “shoulder” months when species have historically shown lower abundances; (3) patterns of critical decline risk—and uncertainty around risk estimates—during high abundance windows might differ between fish predators and their potential suite of prey, and this risk might accumulate at different rates over the next decade as steep population declines in fishes might cause risk to outpace zooplankton taxa; and (4) different regions of the estuary may vary in community‐level risk trends, with variation likely being associated with the longitudinal estuarine gradient (i.e., higher in more variable, seawards regions than in more stable, landwards regions). By examining these questions, we aimed to understand how critical decline risk of estuarine fishes may vary intra‐annually and over space—a critical step to anticipate vulnerability of predator–prey interactions along environmental gradients.

## METHODS

### Fish and plankton surveys

We gathered long‐term monitoring data for fishes and zooplankton in the San Francisco Estuary. For fishes, we used data provided by the California Department of Fish and Wildlife Bay Study (CDFW, 2024, Figure 1). This program has conducted monthly sampling of fishes at fixed stations throughout the estuary since 1980. Fish sampling is conducted using two tow nets: an otter trawl to target benthic species and a midwater trawl to target pelagic species. During each sampling event, captured individuals are counted, identified, and measured. Additionally, sampling effort is quantified to standardize catch metrics. Our analysis focused on age‐0 fishes captured in the midwater trawl. For zooplankton, we used data collected by the Interagency Ecological Program's Environmental Monitoring Program, EMP (CDWR, 2024). The EMP has been sampling zooplankton at fixed stations monthly since 1971, using three types of sampling gear: a macrozooplankton net (505‐μm mesh), a mesozooplankton net (160‐μm mesh), and a teel pump with 43‐μm mesh. In order to target taxa that might be readily consumed by age‐0 forage fishes, we limited our analysis to zooplankton captured in the mesozooplankton net. We filtered each time series to include only monthly surveys after January 1995, as this period maximizes overlap in consistent sampling of fish and zooplankton.

**FIGURE 1:**
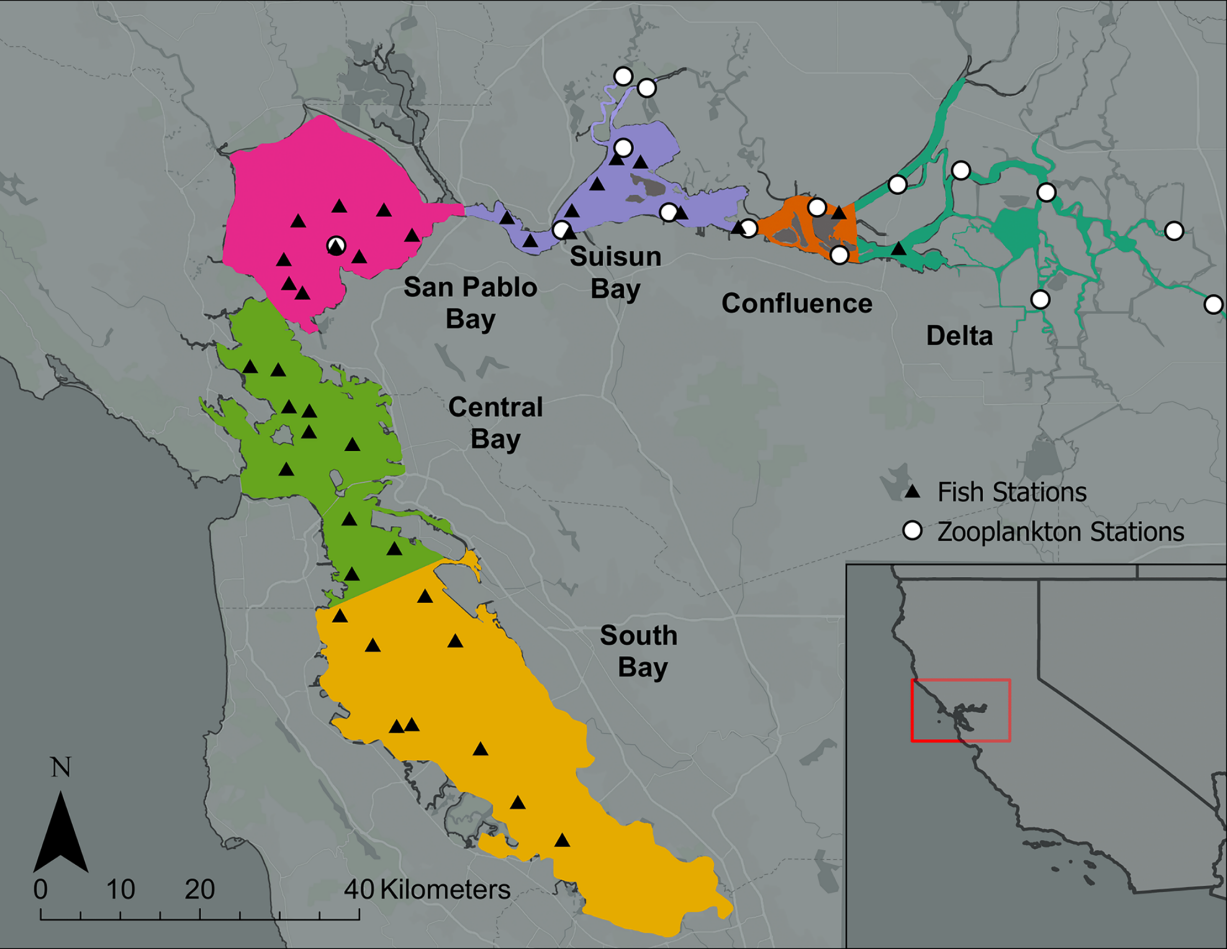
Map of the San Francisco Estuary, California, USA. Regions are shown as color‐coded polygons, with core fish and zooplankton sampling stations shown as black triangles and white circles, respectively. Along the longitudinal axis of the estuarine gradient, and depending on hydroclimatic conditions, salinity can range from polyhaline (18–30 PSU in the Central Bay, which is connected to the Pacific Ocean) to brackish and fresh (0–5 PSU in the Delta).

### Data screening

**FIGURE 2:**
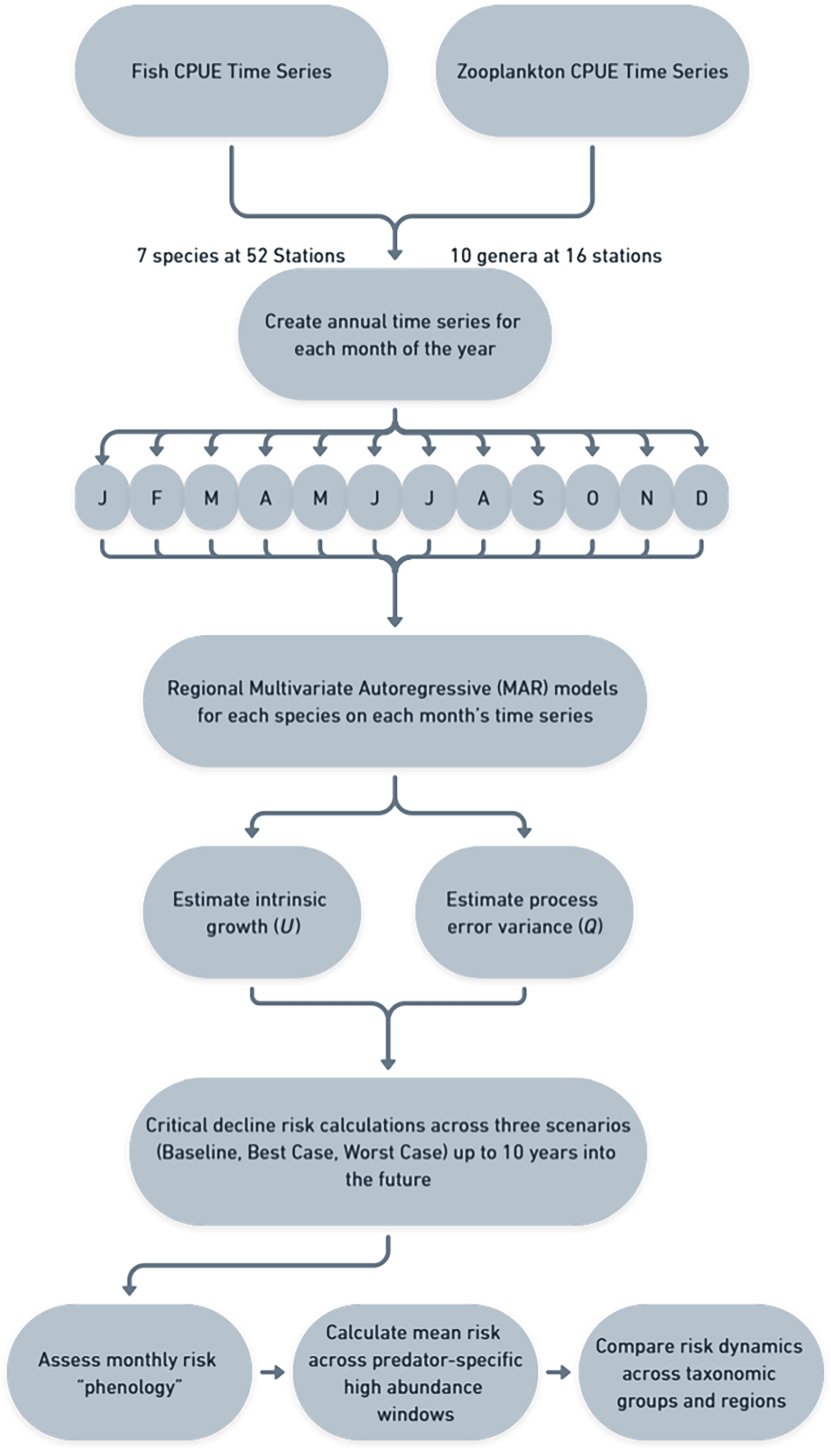
Flow chart of analyses. We illustrate the data inputs, series modeling steps, and critical decline risk outputs.

We assessed data completeness iteratively, to achieve an optimal trade‐off between maximizing data density in the species stations retained and avoiding exclusion of transient or migratory fishes that are only seasonally present in parts of the estuary. We ultimately retained time series with 234 and 50 nonzero detections for zooplankton and fishes, respectively. We then calculated high abundance windows for each fish species by identifying the months that represent most of the mean annual catch for that species in a region (≥80%). In situations where the high abundance windows contained gaps of no more than 1 month, we also included the “skipped” month to obtain an uninterrupted window. This process resulted in seven fish species being retained across 52 stations, and 10 zooplankton genera at 16 stations. Fish and zooplankton stations were assigned to six predefined estuarine regions that have been commonly used for research and management purposes, encompassing from the marine to the freshwater bookend (CDFW, 2024, Colombano et al., 2022; Figure 1). In four of the estuarine regions (i.e., *Delta, Confluence, Suisun, San Pablo*), both fish and potential zooplankton prey were retained. Finally, we broke each year into its constituent months to create 12 annual‐scale, month‐specific time series for each species/station pair (i.e., one time series representing all January data for each year and one representing February data). For subsequent analysis on phenology‐informed risk (see below), we kept all species‐station‐month strata as long as a species was historically present with some regularity (i.e., in ≥30% of the years), achieving higher retention rates than in past work (e.g., Colombano et al., 2022; Pak et al., 2023). Ultimately, we ended up with 155 time series models of fish and 203 of zooplankton, each relevant to a specific taxa, region, and month (Figure 2).

### Time series modeling and risk calculation

With these time series, we fitted Multivariate Autoregressive (MAR) models on catch per unit effort (CPUE) estimates using the R “MARSS” package (Holmes et al., 2014). Here, we used the multivariate structure of MAR models to describe population trajectories within regions. Thus, after grouping the 62 stations into six regions, we built a MAR model for each species‐region‐month stratum. MAR models, unlike state‐space variations of them (e.g., MARSS), do not account for observation error. We acknowledge that observation error is prevalent in biomonitoring data, and metrics‐dependent on process error might be inflated if process error is forced to absorb observation error (Kery et al., 2009; Knape & de Valpine, 2011). However, variation in data density prevented more complex (MARSS) models from converging for some species. To avoid introducing uneven bias across taxa, we opted to apply a consistent MAR modeling framework to all species, rather than correcting for observation error in some but not others. We also examined how parameter estimates changed when observation error was explicitly modeled. These comparisons suggest that overall patterns were generally robust across taxa to the exclusion of observation error (see examination of these trends in Appendix S1: Section S2). We note, however, that although detectability may vary over time and among species (e.g., Huntsman et al., 2022; Sommer et al., 2011), relative trends in abundance can still be informative for management even without explicit correction—especially when monitoring protocols and gear remain consistent (as is the case in the San Francisco Estuary; e.g., Peterson & Barajas, 2018). In this context, we interpret our trend estimates as conservative indicators of relative change between predator and prey rather than absolute abundance (and therefore risk). The general structure of our MAR models, in matrix notation, followed:

(1) $$X_{t}=X_{t-1}+U+W_{t},\;\text{where}\;W_{t}\sim \mathrm{MVN}\left(0,Q\right),$$

where $X_{t}$ is log *x* + 1 transformed CPUE for that species (one time series per station, univariate or multivariate depending on how many stations are represented in that region); *U* captures the long‐term trend of that regional population, and *W*_*t*_ is an error term drawn from a multivariate normal distribution of mean 0 and process error variance/covariance *Q*. For all models, we estimated a single *U* across stations within a region. Similarly, we assumed process error variance to be equal across stations. After estimating *U* and *Q* via maximum‐likelihood (and obtaining 95% CIs for each parameter), we calculated the risk of a species experiencing a 90% population decline (a) over a given time horizon (*T*) up to 10 years from present. This quasi‐extinction probability (*P*_*e*_), referred to throughout as “critical decline risk,” is estimated by using the inverse Gaussian distribution of first passage times for Brownian motion with drift (Dennis et al., 1991; Fieberg & Ellner, 2000; See & Holmes, 2015), where

(2) $$P_{e}=\phi \left(\mu -V\right)+\exp\left(2\mu V\right)\phi \left(-\left(\mu -V\right)\right),$$

(3) $$\mu =-U\sqrt{T/Q},$$

(4) $$V=a/Q\sqrt{T},$$

with ϕ representing the standard normal cumulative distribution function (notation in the above equations have been modified to maintain consistency with MAR parameter notation). Because decline risk can be driven by low population growth potential and/or by high stochasticity, we calculated critical decline risk using across three different scenarios: (1) *baseline risk*, using the maximum‐likelihood estimates for *U* and *Q*; (2) *best case scenario risk*, using the upper end of the bootstrapped CI for *U*, and the lower end of the CI surrounding *Q* (i.e., high growth rate and low process error variance), and (3) *worst case scenario risk*, using the lowest end of the CI surrounding *U* and the highest end of the CI surrounding *Q* (i.e., low or negative intrinsic growth, high process error variance).

### Hypothesis testing

To test the hypothesis that long‐term population trends (*U*) and variability around those trends (*Q*) would vary across fish species, we plotted *U* and *Q* values estimated by MAR models and tested whether systematic differences existed across fish *species* and *regions*, using analysis of variance (ANOVA). We also examined the correlation (Pearson's *R*) between these two parameters to understand whether species with declining trends tended to also be more variable or, if on the contrary, *U* and *Q* varied independently across species and regions.

To test the hypothesis that critical decline risk in a given species would also vary across the year, we assessed the “phenology” of critical decline risk throughout the year for age‐0 forage fishes by plotting monthly risk estimates for each species in each region. We then performed Pearson correlation tests between decline risk and mean population size for each species, pooling data across regions, to assess if these two variables were related—and if so, whether “shoulder” months tended to be safer than months when species have historically shown higher abundances (or vice versa).

To compare how critical decline risk compares between fish (predators) and their suite of zooplankton prey, we examined current critical decline risk during key months of the year (i.e., the months that collectively concentrate 80% of a species' abundance). To this end, we calculated the mean risk for all months identified as high abundance windows for each region. In addition, we calculated the mean decline risk for each zooplankton during the high abundance window of each fish predator—creating paired predator/prey probabilities in each region. Because the age‐0 fishes in our models are capable of eating many or all of the zooplankton in our models (e.g., Jungbluth et al., 2021), we did not make assumptions about prey preferences and instead assumed that each fish species could potentially consume any commonly co‐occurring zooplankton. To assess if fish predator and prey risk during high abundance windows differed, we used ANOVA with “current” critical decline risk (that is, the probability of crossing a 90% decline threshold) as a response variable, and taxonomic *group* (fish or zooplankton) and *region* (if present in more than one region) as predictors. We ran an ANOVA model for each fish species and its paired assemblage of potential zooplankton prey.

To test whether risk is predicted to accumulate at different rates between fish and zooplankton over the next decade, we calculated risk divergence into the future for each fish predator and their set of prey (as previously) across a 10‐year projection. Using analysis of covariance (ANCOVA), we tested whether logit‐transformed risk was explained by taxonomic *group* (fish vs. zooplankton) and/or *region*, using *time* into the future as a covariate (i.e., number of *years*, 1–10). We also considered potential interactions between these terms. A significant interaction between *time* and *group* would indicate a widening (or closing) gap between predator and prey risk—implying mismatch potential. Triple interactions between *time*, *group*, and *region* allowed testing whether diverging gaps in risk between fish and their prey were region‐specific. This analysis also allowed assessing how trends might scale up to the broader regional food webs across the estuary, by modeling risk estimates for the whole community as a function of taxonomic *group*, *time*, *region*, and *fish predator* identity (to cluster individual food webs). Finally, we used the same ANCOVA model structures to estimate the difference in risk between “best case” and “worst case” scenarios. This last analysis allowed for assessing the implications of assuming trends (*U*) and process error (*Q*) on the lower or higher ends that the data supported—and thus the conservation implications of population uncertainty (see Appendix S1: Section S3 for more detail on how we assessed uncertainty through our models).

## RESULTS

### Quantifying population trends and variability

**FIGURE 3:**
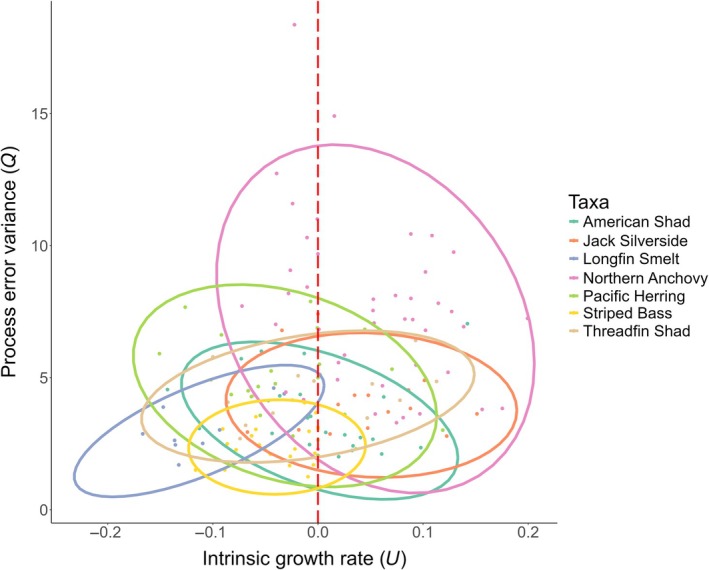
Diversity of population trajectories within and across species. The biplot represents intrinsic growth rates (*U*), versus estimated process error variance (*Q*) estimated by the multivariate autoregressive (MAR) models. For growth rates, values below zero indicate year‐to‐year declines in population estimates for that month and region, while values above zero indicate positive trends. For process error variance, higher values indicate stronger year‐to‐year fluctuations in population estimates for that month and region. Each point represents a species in a given region for each month of the year. We fitted standard ellipses to each species to display the diversity of species‐level trajectories. See Appendix S1: Figure S8 for additional visualizations of these data.

Our models produced a wide range of maximum‐likelihood estimates for fish intrinsic growth rates (long‐term population trends, *U*) and process error variance (variability around these trends, *Q*, Figure 3). Moreover, these variables showed a strong association with each other (Pearson's *R*: 0.207, *p* = 0.009). Specifically, population processes with stronger long‐term growth rates (*U*) also tended to exhibit, overall, higher variability (*Q*). Across the assemblage, we tended to see negative growth rates (median ± SD: −0.003 ± 0.074) that varied by *species* (*F*_6,15.055_, *p* < 0.001). However, positive population trajectories were also possible (positive: 43.1%, negative: 56.8%, range: −0.167 to +0.199). Process error variance, which measures the inherent variability of the population associated with environmental stochasticity, varied strongly by *species* (*F*_6,32.065_, *p* < 0.001), *region* (*F*_5,10.685_, *p* < 0.001), and the interaction between *species* and *region* (*F*_10,3.717_, *p* < 0.001). Notably, Longfin Smelt (*Spirinchus thaleichthys*) displayed nearly ubiquitous declining trajectories and had relatively small levels of process error variance. Conversely, Northern Anchovy (*Engraulis mordax*) showed more positive population growth but did so with very high levels of process error variance (Figure 3). Overall, in agreement with our hypothesis, we observed wide variation in population risk, driven by both spatial variation (regions) and individual species characteristics.

### Phenology of risk

**FIGURE 4:**
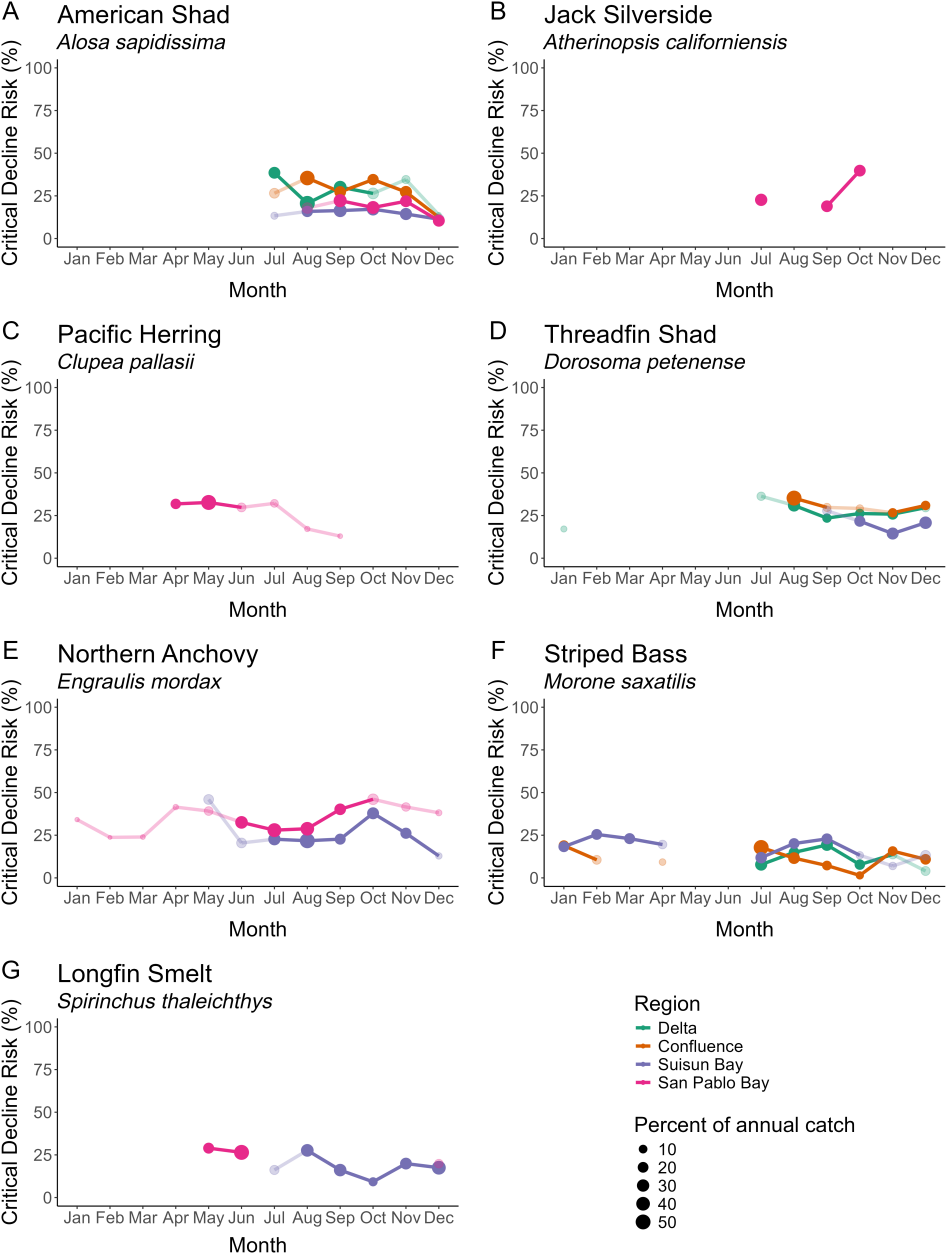
Phenology of risk. Monthly risk that an age‐0 fish species would experience a 90% population decline for that month in each region. Points are scaled by percentage of mean annual catch. High abundance windows, that is, months that contain 80% of the mean annual catch–are in saturated tones while off‐window months are desaturated. Gaps indicate that a species often had zero abundance for that month and region and were thus not modeled.

Fishes showed fluctuating patterns of critical decline risk throughout the year, despite critical decline risks being low overall (mean: 26.8%) (Figure 4). Moreover, taxa in regions at the high end of the salinity gradient (i.e., San Pablo Bay) often were at higher mean risk (San Pablo Bay = 28.3%) than those in lower salinity zones (Delta = 22.2%, Confluence = 20.9%, Suisun Bay = 19.6%). For monthly estimates of risk for all zooplankton taxa, see Appendix S1: Figure S10. We also modeled risk dynamics in the high‐salinity zones of the Central and South Bays and found high mean risk probabilities (Central 36.6%, South 32.1%, Appendix S1: Figure S9). However, as these regions do not have zooplankton monitoring, we excluded them from additional analysis. Notably, no species showed a negative association between monthly risk and abundance–the hypothesized pattern in which species have less risk of critical decline in the months that concentrate more of their relative abundance.

### Implications for trophic dynamics in current and future scenarios

**FIGURE 5:**
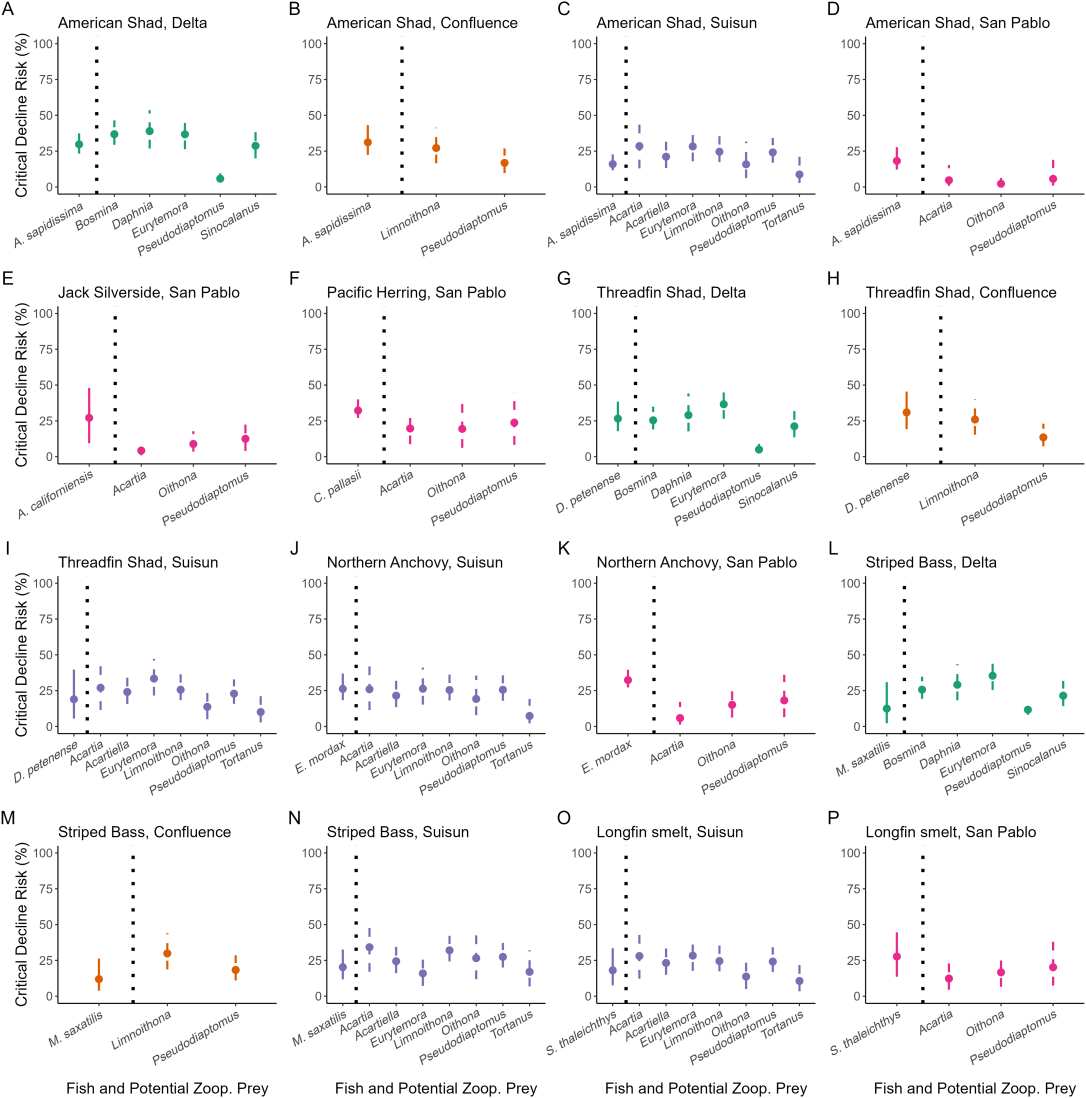
Mean critical decline risk of fish during their high abundance windows paired with the potential suite of zooplankton prey within that same window. Points represent probabilities calculated from maximum‐likelihood parameter estimates. Lower bound represents “best case scenario” wherein decline risks are calculated with the most positive population trend and lowest amount of process error variance. Upper bound represents “worst case scenario” calculated with the most negative population trend and highest amount of process error variance.

We found that critical decline risk was relatively low (mean 21.59%) when we examined paired predator–prey assemblages within their high abundance windows one time step into the future (Figure 5). Though individual zooplankton taxa showed variable patterns of risk in the near term, no fish predator showed significantly divergent decline risk than its corresponding prey assemblage. However, American Shad predator/prey assemblages showed differential patterns by region, with lower critical decline risks associated with San Pablo Bay (*F*_3,4.629_, *p* = 0.002).

**FIGURE 6:**
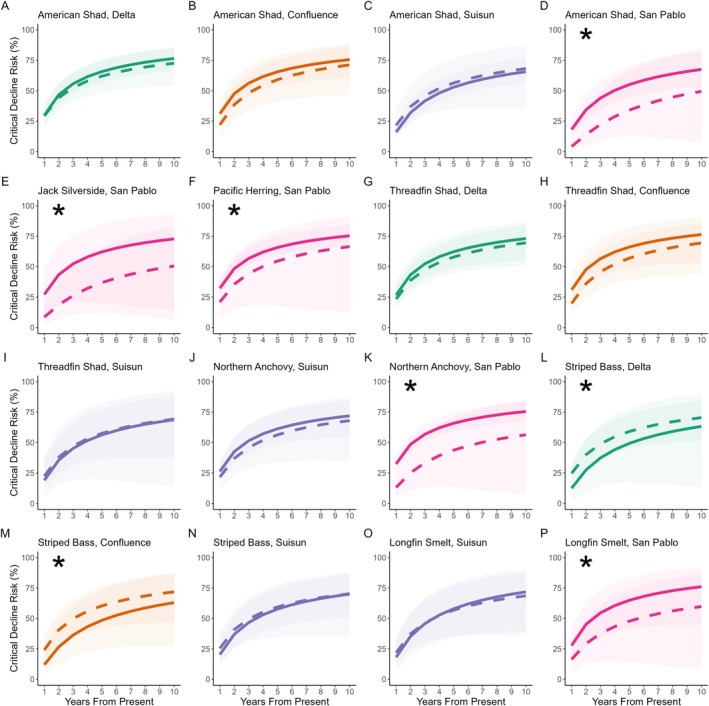
Projection of critical decline risk 10 years into the future. We projected out 10 years from present for fish (solid line) in their high population window and the mean risk of their zooplankton prey assemblage (dashed line) during that same window. Bands represent the range between best case and worst case scenarios. Asterisks represent significant differences between fishes and zooplankton (American Shad, *F*_1,8.721_, *p* = 0.004; Threadfin Shad, *F*_1,3.681_, *p* = 0.057; Jack Silverside, *F*_1,52.744_, *p* < 0.001; Pacific Herring, *F*_1,19.192_, *p* < 0.01; Northern Anchovy, *F*_1,22.016_, *p* < 0.001; Striped Bass, *F*_1,22.117_, *p* < 0.001; Longfin Smelt, *F*_1,10.652_, *p* = 0.0015).

Despite this “balanced” risk between fish and their prey currently, as we projected critical decline risk into the next decade, we found widespread divergence between fishes and their zooplankton prey across different regions of the estuary (*F*_3,33.265_, *p* < 0.001 for the group by region interaction; see next section for community‐level trends). Among individual fishes, all except the Threadfin Shad displayed differential risk compared to their prey assemblages (Figure 6), but this often varied by region. Throughout the 10‐year projection, we also found strong regional differences in American Shad (*F*_3,48.51_, *p* < 0.001), Northern Anchovy (*F*_1,22.671_, *p* < 0.001), and Longfin Smelt food webs (*F*_1,10.557_, *p* = 0.001), as well as *group* by *region* interactions for all three of these fish predators (American Shad *F*_3,8.321_, *p* < 0.001; Northern Anchovy *F*_1,12.746_, *p* = 0.02; Longfin Smelt *F*_1,13.563_, *p* < 0.001). Overall, we saw support for our hypothesis that fishes display higher levels of risk than their zooplankton prey into the future, but these estuary‐level trends are a function of dynamics across all sub regions and predominantly driven by strong divergence in San Pablo Bay.

### Community‐scale observations

**FIGURE 7:**
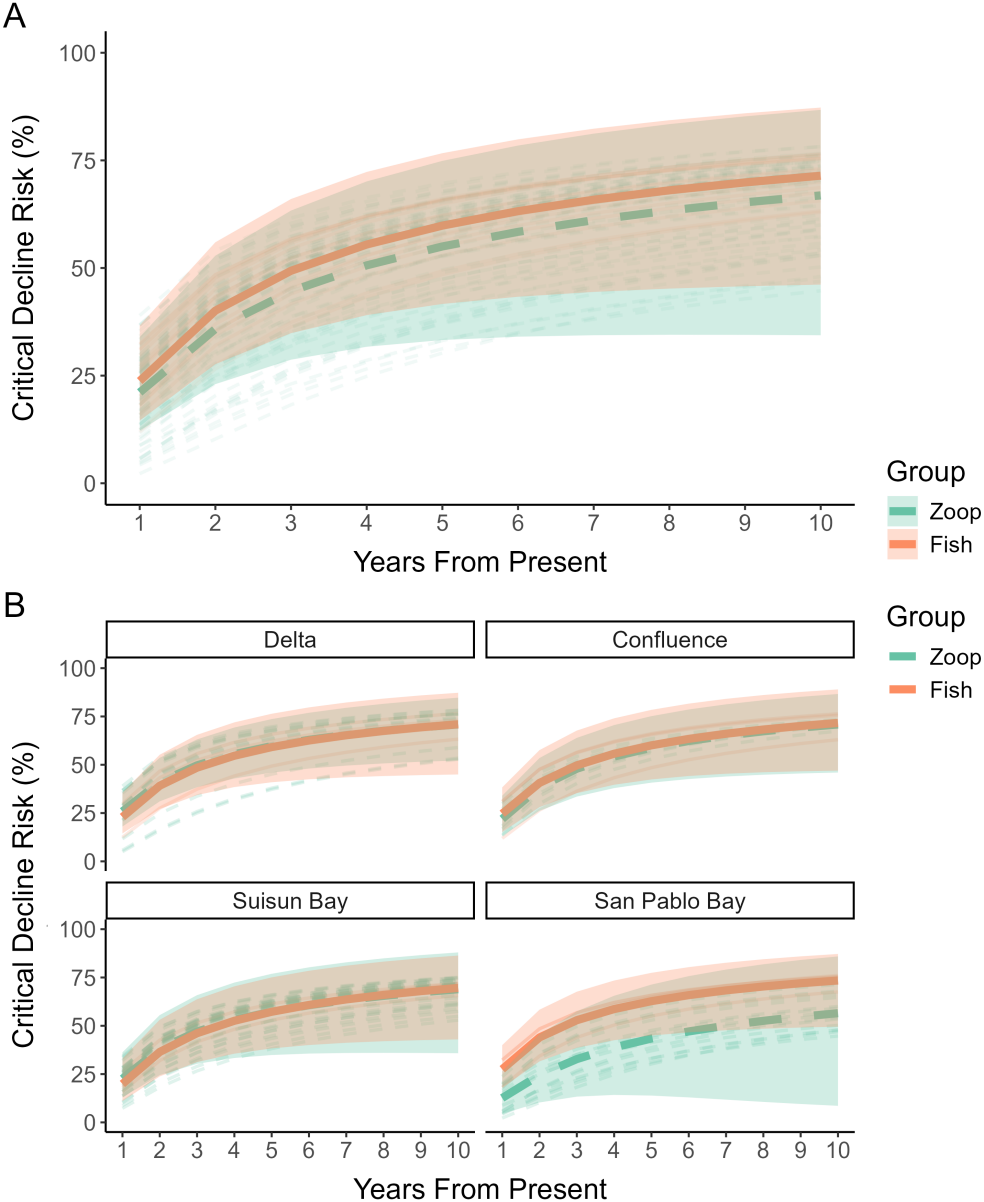
Accumulation of community‐wide risk across regions of the estuary. We display mean decline risk for all predators (orange, solid line) and their paired prey assemblages (green, dashed line) in each region during high abundance windows projected out for 10 years. Individual taxa are represented by desaturated lines. Bands represent the mean range between best case and worst case scenarios (see *Methods* for details).

Divergences between fish and zooplankton critical decline risks also manifested at the community level, with zooplankton assemblages throughout the estuary having lower mean decline risk than fishes (*F*_1,29.458_, *p* < 0.01). However, these differences are predominantly driven by patterns in San Pablo Bay (Region effect *F*_3,57.025_, *p* < 0.001, Figure 7). Moreover, we found the rate of divergence in trends between groups was unique to each region of the estuary (*F*_3,32.268_, *p* = 0.006 for the *group by region* interaction), as well as unique to each predator/prey assemblage (*F*_6,9.291_, *p* < 0.01 for *fish predator identity*). Despite the higher baseline critical decline risk in fish assemblages, uncertainty (i.e., the difference between risk from best case and worst case scenario projections) was higher for zooplankton relative to fish (*F*_1,33.688_, *p* < 0.001) and accumulated differently between trophic groups (*F*_1,11.054_, *p* < 0.001 for the *group* by *time* interaction). Moreover, uncertainty varied by *region* (*F*_3,100.639_, *p* < 0.001), and *groups* in each *region* accumulated risk differentially through time (*F*_3,7.763_, *p* < 0.001 for the triple interaction between *group, time*, and *region*). This interaction indicates that a wider range of critical decline outcomes are possible for zooplankton across the estuary. Overall, these results suggest that patterns of risk can scale up from individual predator/prey assemblages to the community level. However, local conditions—notably the salinity gradient—control how these patterns might manifest into the future. For additional exploration and visualization of our results, see the companion app “Fish‐Zooplankton Risk Explorer” https://baydelta.shinyapps.io/Fish‐Zooplankton‐Risk‐Explorer/.

## DISCUSSION

Conservation scientists often quantify extinction risk to triage populations and prioritize the allocation of limited resources. However, these estimates often assume that decline risk is consistent within relatively small temporal and spatial scales (Coulson et al., 2001). Moreover, PVAs are usually explored within the context of a single species and assessments of risk across food webs are comparatively rare (Sabo, 2008). Here, we sought to evaluate how critical decline risk for age‐0 forage fishes and their potential prey varies throughout the year across the San Francisco Estuary. Both forage fishes and their zooplankton prey were characterized by periods of concentrated abundance within years, emphasizing the importance of considering decline risk at sub‐yearly scales. We found that many focal fish species showed negative population growth and that critical decline risk was variable across months within the first year of life. Additionally, we found that predator and prey decline risk diverged across a 10‐year projection, suggesting a potential for future trophic mismatches. However, these divergent patterns were most pronounced in one region of the estuary (San Pablo Bay), indicating that local environmental factors might drive disruptions to the food web. Our findings underscore the need to consider fine‐scale temporal and spatial variation in risk in estuarine taxa. The observed widening gaps in risk and uncertainty around risk between trophic levels advance the notion that phenological shifts and associated trophic mismatches are an emergent, yet largely underappreciated consequence of global change on ecological communities (Cohen et al., 2018; Fournier et al., 2024).

### Widely declining fish population trajectories

There is growing evidence that the capacity of the San Francisco Estuary to support forage fish populations has substantially diminished in recent decades (Rosenfield & Baxter, 2007). The resulting pelagic organism decline has been marked by dramatic collapses in forage fish populations (Sommer et al., 2007), and these declining abundances are reflected across our MAR models (Figure 3). Previous applications of time series modeling in this system have identified strong negative trends at the population level driven by a variety of environmental factors, including water clarity and the variable position of the 2‰ isohaline zone, *X*_2_ (Mac Nally et al., 2010). Understanding the causes and consequences of these declines requires linking abundance trends to probabilistic estimates of extinction outcomes.

Our critical decline risk calculations are based on two primary measures of population dynamics: intrinsic growth rates and process‐driven variability. Over half of all fish time series (56.8%) displayed negative intrinsic growth rates, commonly resulting in increased critical decline risk—a high near‐term probability that the species will no longer be present, that month, in that region of the estuary. Notably, the Longfin Smelt, a native osmerid that was historically abundant in the San Francisco Estuary (Tempel et al., 2021), exhibited nearly ubiquitous patterns of negative growth across regions and months (Figure 3). Declines of Longfin Smelt are well established (Nobriga & Rosenfield, 2016), and habitat degradation and successive recruitment failures have led to its recent Federal listing under the Endangered Species Act (USFWS, 2024). In this case, the strongly negative growth rates identified by our models likely drive the observed patterns of critical decline risk. Conversely, taxa with highly variable population dynamics can still exhibit high decline risk even with positive growth trends. For instance, the Northern Anchovy often maintained positive growth rates but did so in a highly variable manner (Figure 3). This predominantly marine species opportunistically uses estuarine habitats (Allen et al., 2006). Populations tend to show boom‐and‐bust dynamics, with recruitment often tied to lower delta outflow, and juvenile abundance positively correlating with drought conditions that increase system salinity (Colombano et al., 2022). A strong reliance on environmental stochasticity to maintain abundance levels is reflected in high population variance, increasing the likelihood of a critical decline event. Overall, both the overall negative growth rates and high population variance identified by our time series models illustrate an assemblage in flux.

### Phenology of risk

Fish recruitment to adult life stages (and fisheries) is often highly sensitive to fluctuations in early life survivorship (Fournier et al., 2021; Hjort, 1914; Winemiller & Rose, 1992), and our models revealed that age‐0 critical decline risk was not uniform across months. The dynamic environmental conditions of estuaries can disproportionately impact juvenile fishes that rear in these nursery habitats (Jenkins et al., 2022; Morrongiello et al., 2014). For example, seasonal changes in delta outflow and salinity can alter resource availability during key growth periods (Reis et al., 2019). Additionally, widespread anthropogenic alteration of breeding and rearing habitats throughout the San Francisco Estuary has negatively affected early life stages of estuarine fishes (Cloern & Jassby, 2012). Despite our expectation that critical decline risk would be higher during months with historically low abundances, we did not observe that pattern and found that critical decline risk was similar across high abundance and low abundance windows for age‐0 fishes. Low juvenile survivorship is often offset by increased fecundity, which helps to buffer mortality (Winemiller & Rose, 1993) and likely explains the observed risk patterns. Moreover, density‐dependent resource exploitation during high abundance windows might affect population performance more acutely than density‐independent factors in other parts of the growth season (DeAngelis et al., 1993).

Spawning and hatching are key phenological events, the timing of which tends to be highly sensitive to environmental change (Hovel et al., 2017; Lawrence et al., 1997). Our analyses and risk estimates focused on these high abundance windows. If environmental conditions during these periods become unsuitable, estuarine taxa might respond by advancing or delaying their phenology (Asch et al., 2019; Chevillot et al., 2017). However, estuarine fishes might be limited in their abilities to phenologically track changing environmental conditions long‐term (Fournier et al., 2024). Moreover, desynchronization between a population and its key resource base can destabilize food webs (Stenseth & Mysterud, 2002; Zhemchuzhnikov et al., 2021). Our results highlight the need to closely monitor decline risk during the key periods when a species is present, as assuming risk consistency could mislead managers to overlook potentially impactful moments of heightened risk.

### A widening gap in risk, and risk uncertainty, between fishes and their prey

While PVA are generally considered within single species contexts, we sought to pair fish predators with their potential prey assemblage during important phenological windows. Though near‐term patterns of decline risk were similar between predators and their prey, we observed that risk diverged when considering a 10‐year time horizon. Predators and prey often fluctuate together in lagged cycles (Chesson, 1978) and typically reach equilibrium over evolutionary timescales (Smith & Slatkin, 1973). While predictable disturbances can stabilize trophic interactions (Vasseur & Fox, 2009), strong environmental fluctuations driven by global change might disrupt these relationships (Bretagnolle & Gillis, 2010). Additionally, phenological shifts that decouple historically synchronous species can cause trophic mismatches that destabilize food webs (Thakur, 2020; Varpe & Fiksen, 2010), and phenological trends between estuarine predators and prey could be diverging (Fournier et al., 2024).

Our projections indicated that fishes often exhibited higher rates of decline than their prey over a 10‐year period. If predators are extirpated during key phenological windows while their prey persist, the resulting trophic release of zooplankton taxa might increase the likelihood of harmful algal blooms directly (depending on the zooplankter) or indirectly through cascading phytoplankton release (Jachowski et al., 2020). Conversely, Striped Bass, a non‐native but well‐established species in the San Francisco Estuary, often showed lower critical decline risk than the prey taxa it consumes. Despite its high diet adaptability (Young et al., 2022), significant declines in prey assemblages during key phenological periods might still result in population declines (Nobriga & Feyrer, 2008). Indeed, altered patterns of prey availability are thought to be a significant driver of the estuary's POD (Sommer et al., 2007). Importantly, we observed different levels of risk uncertainty (i.e., the difference between best case and worst case scenarios) between fishes and zooplankton throughout the estuary. In general, zooplankton exhibited a wider range of possible trajectories than fishes. The fast generation times and boom‐and‐bust cycles characteristic of many zooplankton taxa, including the widespread genera examined in our model, complicate precise estimates of decline risk (Lane, 1975). Moreover, biotic controls on plankton populations not assessed by our models (e.g., grazing pressure by invasive bivalves, Nichols et al., 1990) might further destabilize zooplankton prey pools. Thus, while the aggregate stability of the assemblage might facilitate prey‐switching (Potts et al., 2016), large‐scale collapses predicted by our “worst case” models would likely destabilize whole food webs.

In our study, we found that the strongly divergent trends (i.e., “widening risk gaps” between fishes and their prey) occurred in a single region of the estuary, San Pablo Bay. This region is near the Pacific Ocean and has the highest salinity levels in the study area. Consequently, long‐term droughts that shift the salinity gradient upriver, coupled with the influence of recent marine heatwaves, might make this region especially vulnerable to hydroclimatic fluctuations (Sanford et al., 2019). As fishes and plankton have variable tolerances to environmental conditions such as increased temperature or salinity (Beitinger et al., 2000; Qasim et al., 1972), harsher conditions in this region might differentially influence decline risk. Although individual taxa might display variable levels of risk, a diversity of trends might promote community‐level stability (Ovaskainen et al., 2013). At the estuary‐wide scale, the taxonomic richness of forage fishes appears to facilitate portfolio effects that enhance community resilience despite declines of individual taxa (Colombano et al., 2022). Additionally, spatial insurance may allow individual subregions to bolster the metapopulation when local conditions become unsuitable elsewhere. Indeed, previous time series analyses have been used to infer metapopulation structure and identify subpopulations that disproportionately impact regional decline risk (Sarremejane et al., 2021; Ward et al., 2010). Similarly, except for Striped Bass, the divergent patterns we observed in San Pablo Bay appear to be localized, as other subregions throughout the estuary show similar risk for fishes and their prey. Thus, management strategies that either mitigate risk in San Pablo Bay, or enhance the capacity of other subregions to serve as refuge habitats, might confer food‐web stability throughout the region.

### Limitations and future directions

The increasing availability of biological monitoring data makes time series‐based approaches a suitable alternative to traditional, mechanistic PVA (Chaudhary & Oli, 2020; Holmes et al., 2007). Here, we leveraged long‐term monitoring data for age‐0 forage fishes and their zooplankton prey in the San Francisco Estuary to assess critical decline risk across multiple scenarios. Nonetheless, these methodologies have limitations. First, we explored sub‐annual trends by breaking the time series into its constituent months. While this fine‐scale analysis revealed when in a given season a particular fish may be more likely to disappear, this approach assumes that seasonal patterns remain consistent across years—an assumption that does not account for the high interannual variability in estuaries. Other commonly used parametric time series approaches, such as MAR/MARSS models with seasonal fixed effects or seasonal ARIMAs, would face similar limitations, as they also assume seasonal stationarity. More flexible methods, such as time‐frequency approaches (e.g., wavelets) or nonparametric models (e.g., GAMs), could address this issue but come with trade‐offs. For instance, wavelet approaches would require vastly higher frequency sampling than biomonitoring programs (like those we used) provide, and nonparametric models tend to lack the process‐based parameters needed for PVA. Another key consideration is that our month‐specific approach effectively partitions a single time series into 12 separate series, thereby limiting the influence of temporal autocorrelation. Similar strategies are commonly applied in hydrology and other physical sciences to understand whether particular months or seasons are more sensitive to environmental processes (Chalise et al., 2021; Cloern, 2019; Testa et al., 2018). Given that estuarine systems respond dynamically to environmental drivers, methods that allow for seasonal flexibility in ecological processes are appropriate. Second, our critical decline metrics might be inflated because they are derived solely from process error while observation error is not assessed (Knape & de Valpine, 2011). However, these estimates remain valuable because our modeling approach predominantly examines trend divergence over the absolute magnitude of risk to ameliorate any potential bias inconsistency across fish species and their potential prey (but see Appendix S1: Section S2). Furthermore, the probability of not detecting a species in a given month (i.e., “extinction” from an observation standpoint) is still critical, as it may prompt management and conservation actions, even if the species was not detected due to imperfect observation rather than true absence (e.g., Delta Smelt, Rose et al., 2013). Third, direct biotic interactions are difficult to estimate. We sought to ameliorate this shortcoming by calculating average risk of prey within phenological windows bespoke to each fish species. However, these indirect methods might not fully assess increases in decline risk due to prey availability and altered prey pools are predicted to influence pelagic organism population collapses (Cloern & Jassby, 2012; Sommer et al., 2007). The age‐0 fishes included in our models are capable of consuming many of the common zooplankton prey species found within the estuary (Howe et al., 2014; Jungbluth et al., 2021; Nobriga & Feyrer, 2008). Though these species might exhibit varying levels of prey selectivity among the zooplankton in our models (Nobriga & Feyrer, 2008), high‐resolution diet data were not available for all fish species. Thus, we did not make any assumptions about prey preferences. While this approach suffices to quantify mismatch potential, further studies directly linking predators and their specific prey by assessing species‐specific predator preferences and prey vulnerability would greatly improve our ability to constrain realized (as opposed to potential) trophic mismatches. Finally, our models examined spatial dynamics by assessing decline risk at subregional scales. In doing so, we found that San Pablo Bay has elevated mismatch risk relative to other regions of the estuary. As migration through the estuary is common for many of our focal taxa, explicit examinations of metapopulation structure might enhance our understanding of whole‐estuary ecological dynamics.

### Concluding remarks

The richness and complex life cycles of estuarine biota often complicate management and conservation efforts in these dynamic ecosystems (Jha et al., 2008; Lauchlan & Nagelkerken, 2020). Here, we used a novel quantitative approach to estimate critical decline risk in a community context. Contrary to what is typically assumed, we found that even within a given species and life stage, critical decline risk can be highly variable across months of the year–suggesting high potential for climate‐induced trophic mismatches (Visser, 2022). Because predator–prey dynamics and food limitation have been linked to the ongoing fish population declines in the San Francisco Estuary (Baxter et al., 2008; Cloern & Jassby, 2012), our results are directly relevant to conservation efforts in this system. Notably, our approach could be transferred to other estuaries with similar long‐term datasets on species abundances across trophic levels. A more robust understanding of fine‐scale temporal dynamics within food webs should help design more effective conservation strategies for vulnerable populations undergoing climate‐change‐induced phenological shifts.

## CONFLICT OF INTEREST STATEMENT

The authors declare no conflicts of interest.

## Supporting information


Appendix S1.

## Data Availability

Data and metadata (Fournier et al., 2025a) are available in Dryad at https://doi.org/10.5061/dryad.4j0zpc8nd. Code (Fournier et al., 2025b) is available in Zenodo at https://doi.org/10.5281/zenodo.13800013. Raw abundance time series data (CDFW Bay Study and IEP Environmental Monitoring Program Zooplankton Study) were accessed via the California Department of Fish and Wildlife and California Department of Water Resources. For additional exploration and visualization of the results, see the companion app “Fish‐Zooplankton Risk Explorer” at https://baydelta.shinyapps.io/Fish‐Zooplankton‐Risk‐Explorer/.
