# Supplementary material for: Phenology‐informed decline risk of estuarine fishes and their prey suggests potential for future trophic mismatches
Source: Ecol Appl. 2025 Nov 10;35(7):e70130. doi: 10.1002/eap.70130 (PMC12602011; doi:10.1002/eap.70130)
Supplement: Supplementary file 1 — Appendix S1. [file EAP-35-e70130-s001.pdf]

Appendix S1

Phenology-informed decline risk of estuarine fishes and their prey suggests potential for future trophic mismatches

Robert J. Fournier, Tyler C. Marino, Stephanie M. Carlson, Albert Ruhí

Ecological Applications

Section S1: Supplementary Tables and Figures

Table S1: List of modeled taxa

Taxa	Group	Common name
Dorosoma petenense	Fish	Threadfin shad
Morone saxatilis	Fish	Striped bass
Spirinchus thaleichthys	Fish	Longfin smelt
Alosa sapidissima	Fish	American shad
Clupea pallasii	Fish	Pacific herring
Atherinopsis californiensis	Fish	Jack silverside
Engraulis mordax	Fish	Northern anchovy
Tortanus	Zooplankton	NA
Oithona	Zooplankton	NA
Acartia	Zooplankton	NA
Daphnia	Zooplankton	NA
Bosmina	Zooplankton	NA
Sinocalanus	Zooplankton	NA
Eurytemora	Zooplankton	NA
Pseudodiaptomus	Zooplankton	NA
Limnoithona	Zooplankton	NA
Acartiella	Zooplankton	NA

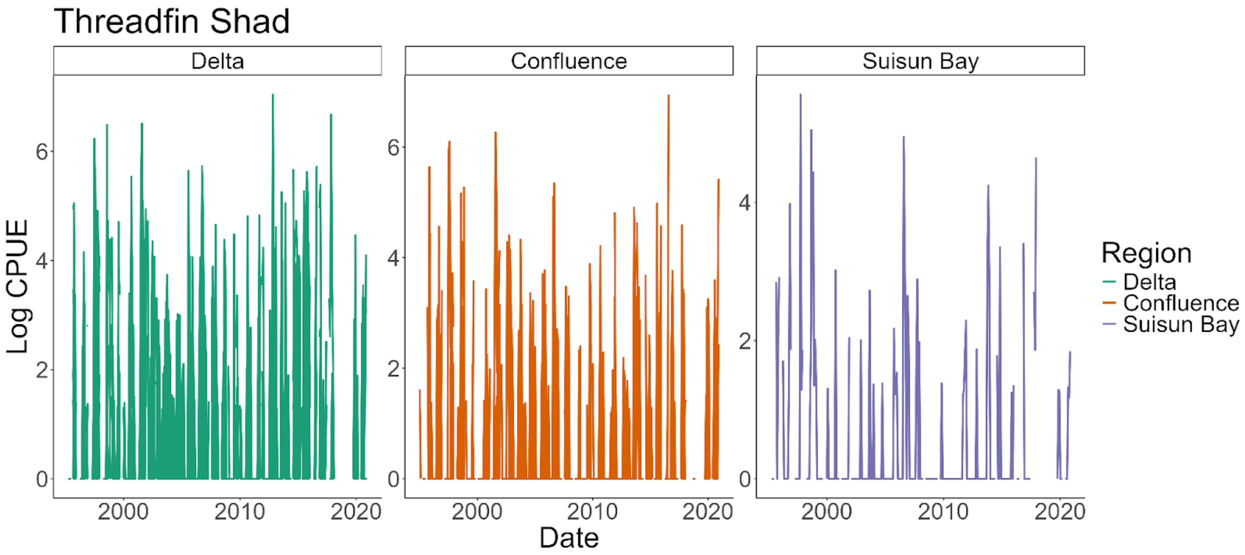

Figure S1: Time series of adjusted CPUE for Threadfin Shad.

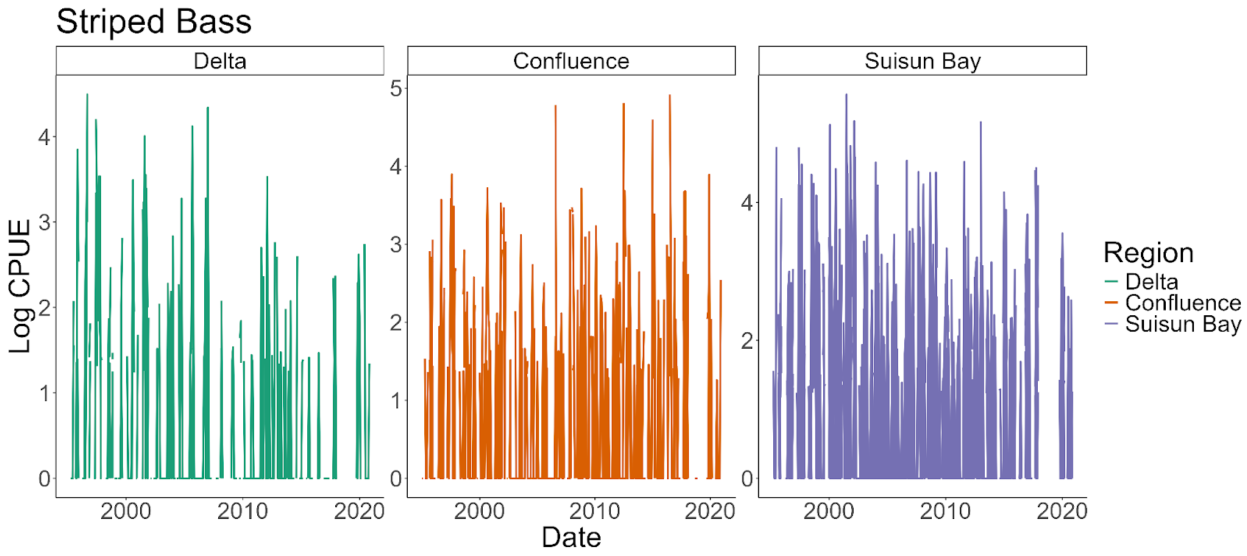

Figure S2: Time series of adjusted CPUE for Striped Bass

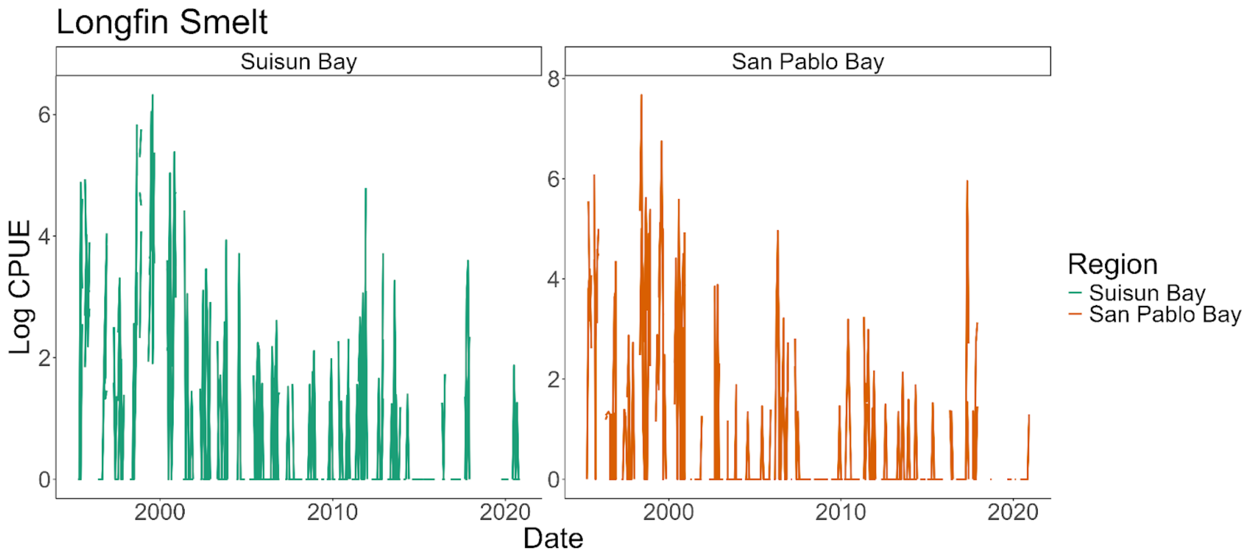

Figure S3: Time series of adjusted CPUE for Longfin Smelt.

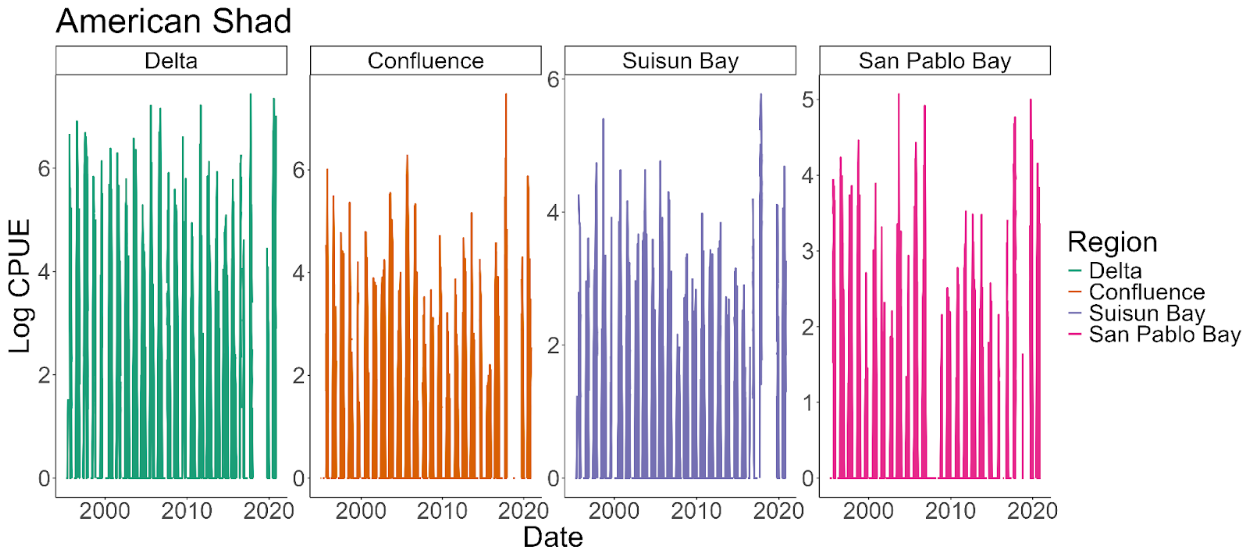

Figure S4: Time series of adjusted CPUE for American Shad.

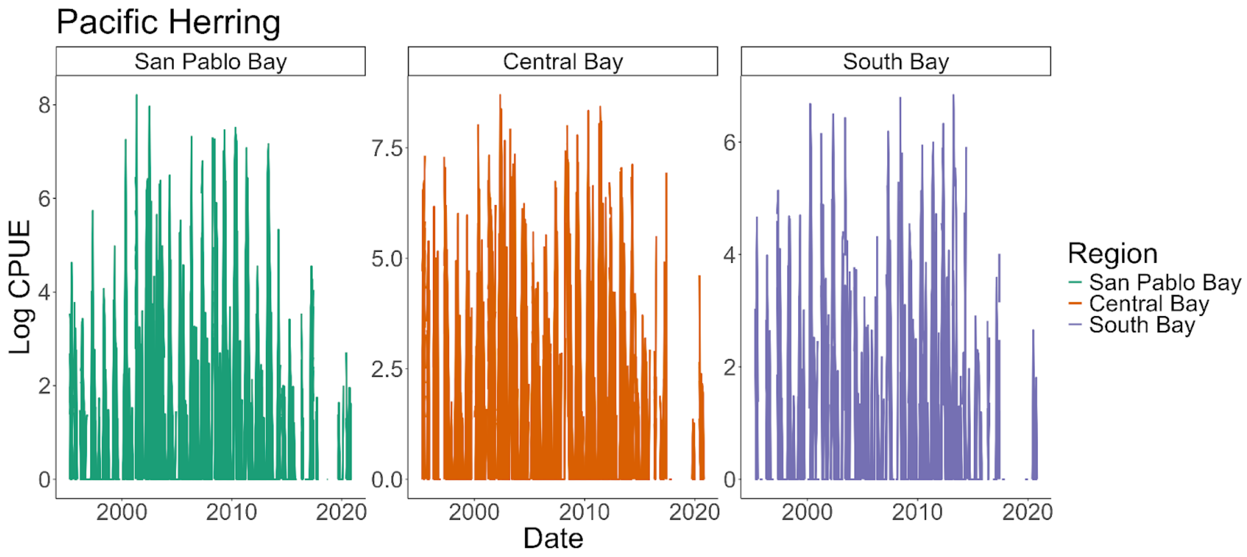

Figure S5: Time series of adjusted CPUE for Pacific Herring.

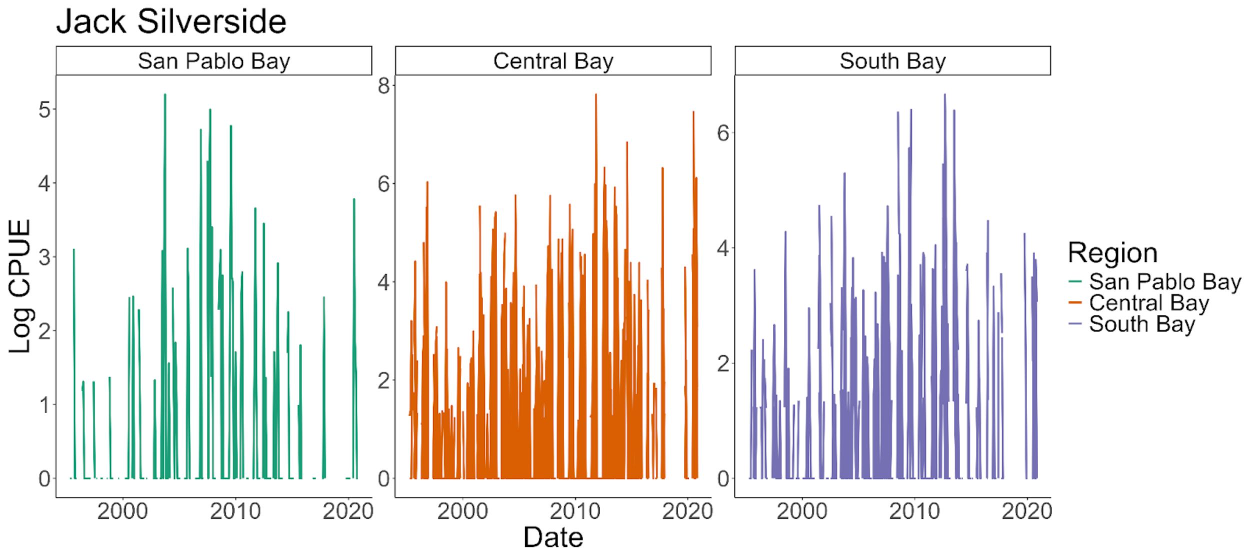

Figure S6: Time series of adjusted CPUE for Jack Silverside.

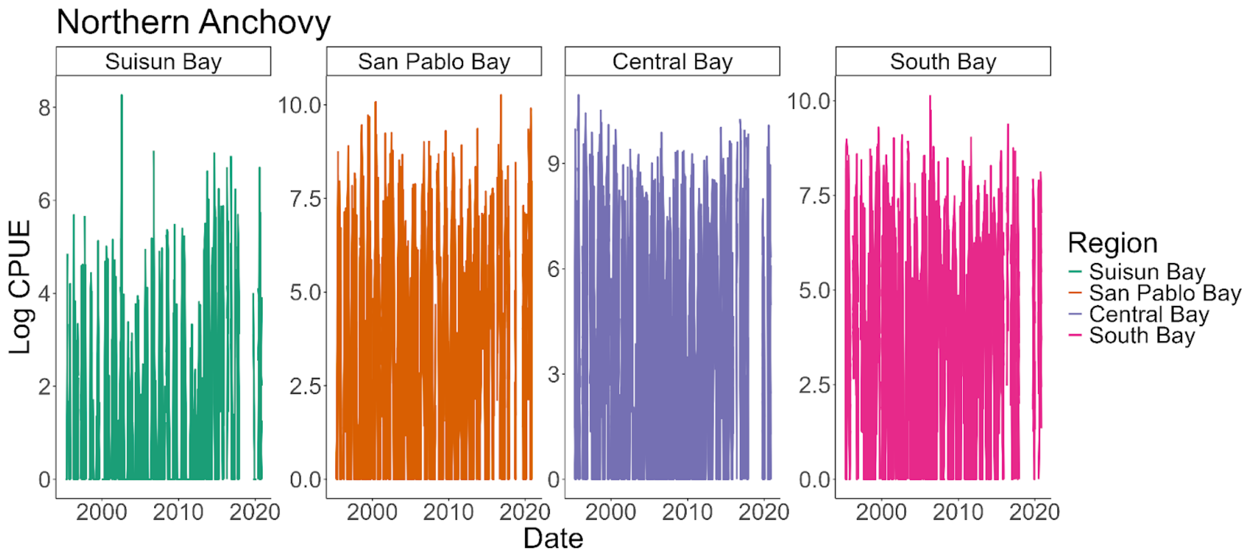

Figure S7: Time series of adjusted CPUE for Northern Anchovy.

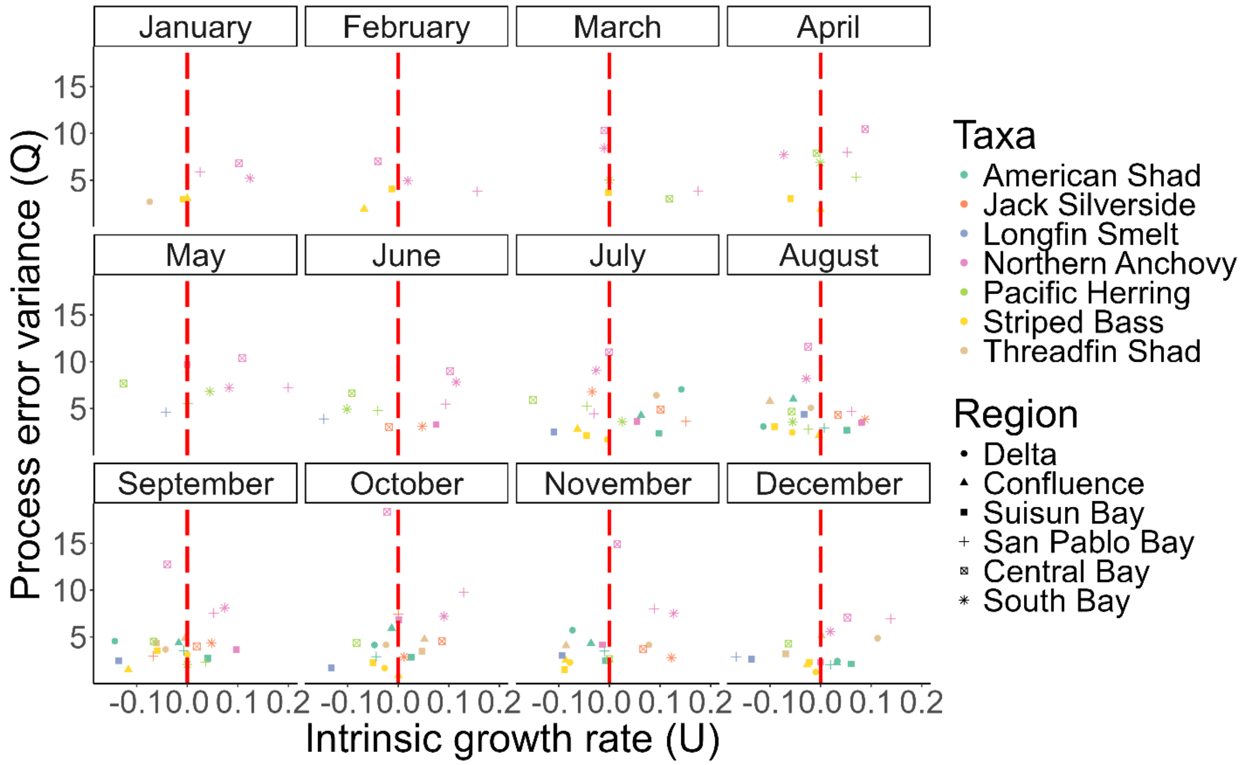

Figure S8: Intrinsic growth rates (*U*), versus estimated process error variance (*Q*) estimated by the Multivariate Autoregressive (MAR) models. For growth rates, values below zero indicate year-to-year declines in population estimates for that month and region, while values above zero indicate positive trends. For process error variance, higher values indicate stronger year-to-year fluctuations in population estimates for that month and region. Values are broken down by month. Colors represent fish taxa and point shape represents region.

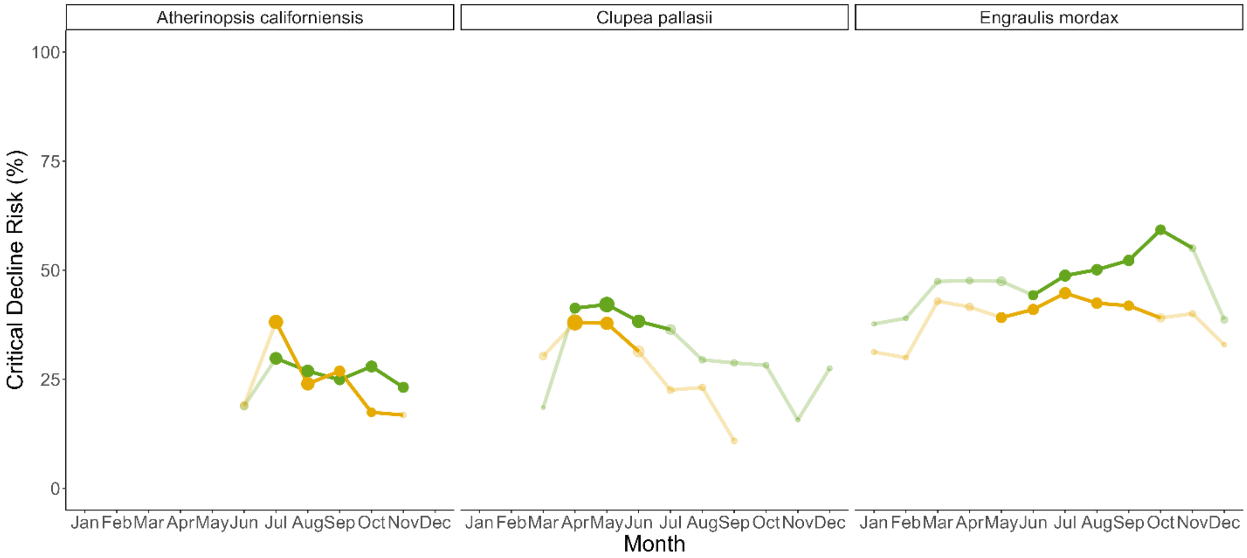

Figure S9: Monthly risk that an age-0 fish species would experience a 90% population decline for that month in the Central and South Bay regions. Points are scaled by percentage of mean annual catch. Key windows–i.e., months that contain 80% of the mean annual catch–are in saturated tones while off-window months are desaturated. Gaps indicate that a species often had zero abundance for that month and region and were thus not modeled.

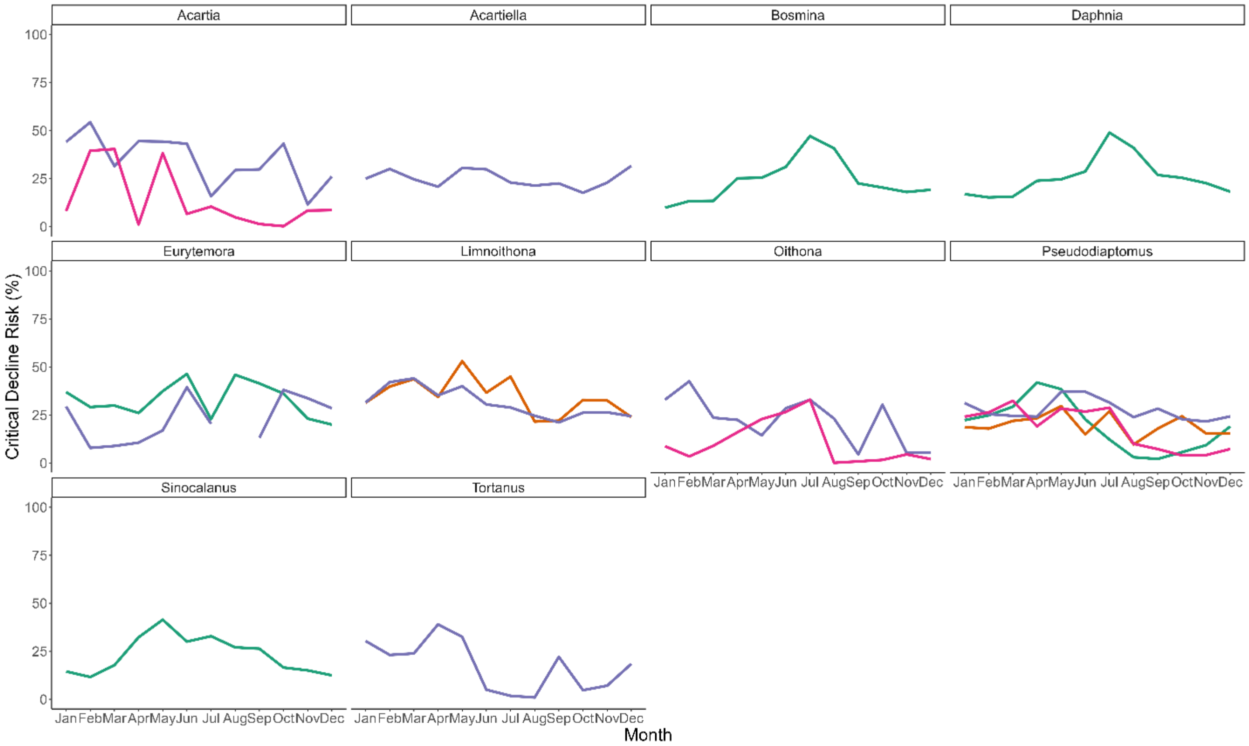

Figure S10: Monthly risk that a given zooplankton taxa would experience a 90% population decline for that month in each region. Gaps indicate that a species often had zero abundance for that month and region and were thus not modeled.

Section S2: Exploration of the influence of observation error on parameter estimates.

In the main manuscript, we present results from MAR models that do not incorporate observation error, primarily due to challenges fitting appropriate state-space models to the data. However, given the importance of observation error in analyses based on biomonitoring data, we sought to assess whether its exclusion meaningfully influenced parameter estimates. To do so, we constructed MARSS models for each species/region/month stratum. We assumed consistent observation error across stations within each month/region stratum and made no assumptions about latent state groupings to improve convergence. We then compared the MAR and MARSS model estimates for intrinsic growth rates (U) and process error variance (Q) (Figure S11).

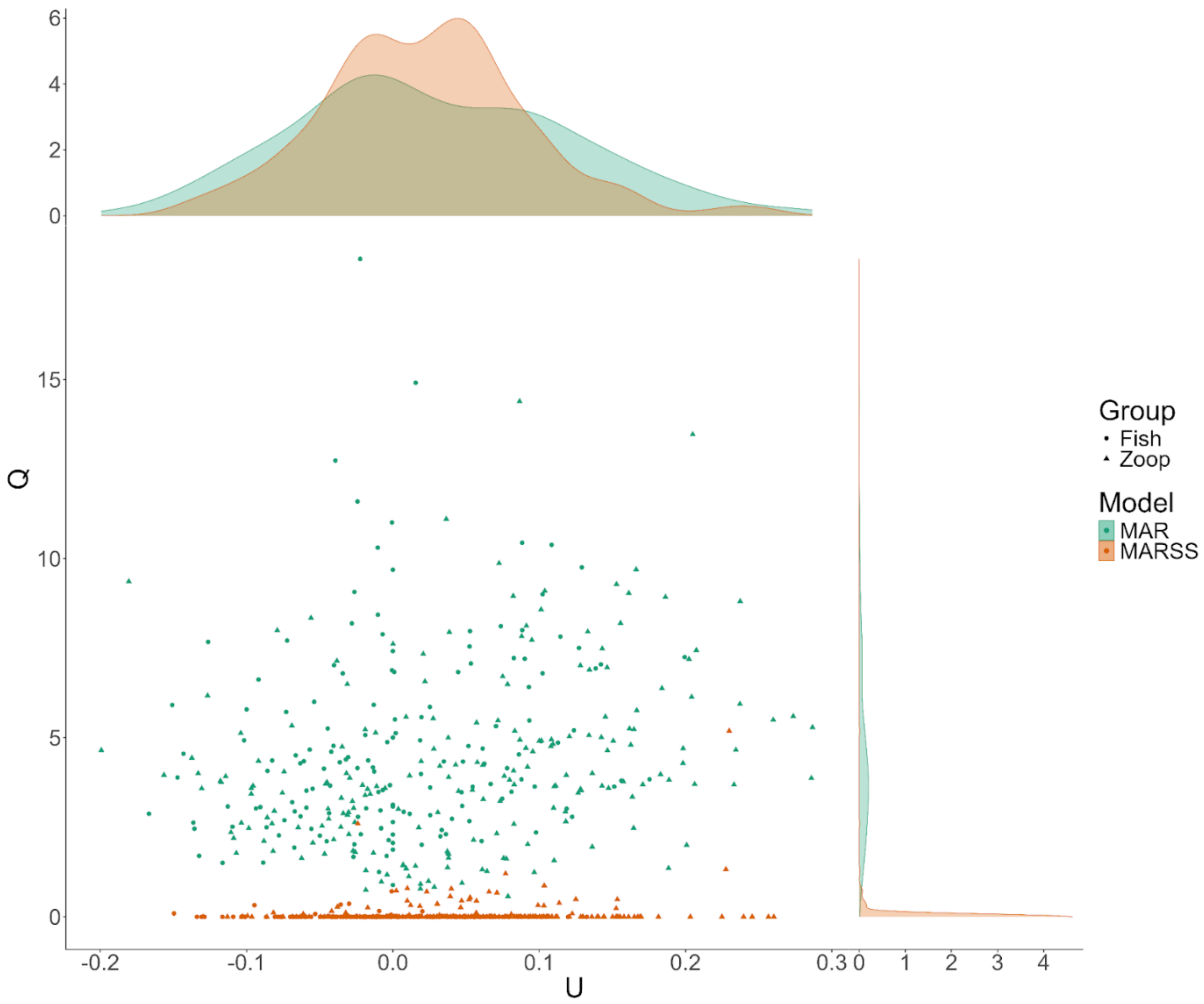

Figure S11. Comparison of MAR vs MARSS model estimates for process error variance (Q) and intrinsic growth rates (U).

We found that when attempting MARSS, nearly all models (97%, 256 out of 265 fish models) had convergence issues. Most frequently (84% of the time), the process error variance (Q) collapsed to zero, and all variation was absorbed by the observation error variance term (R). This is a common symptom of overfitting or model degeneracy, even when ML optimization formally converges (Holmes et al. 2014). To evaluate the impact on our parameter estimates, we compared MAR and MARSS estimates of U across all species, months, and regions in a 1:1 plot (Fig. S12, next page).

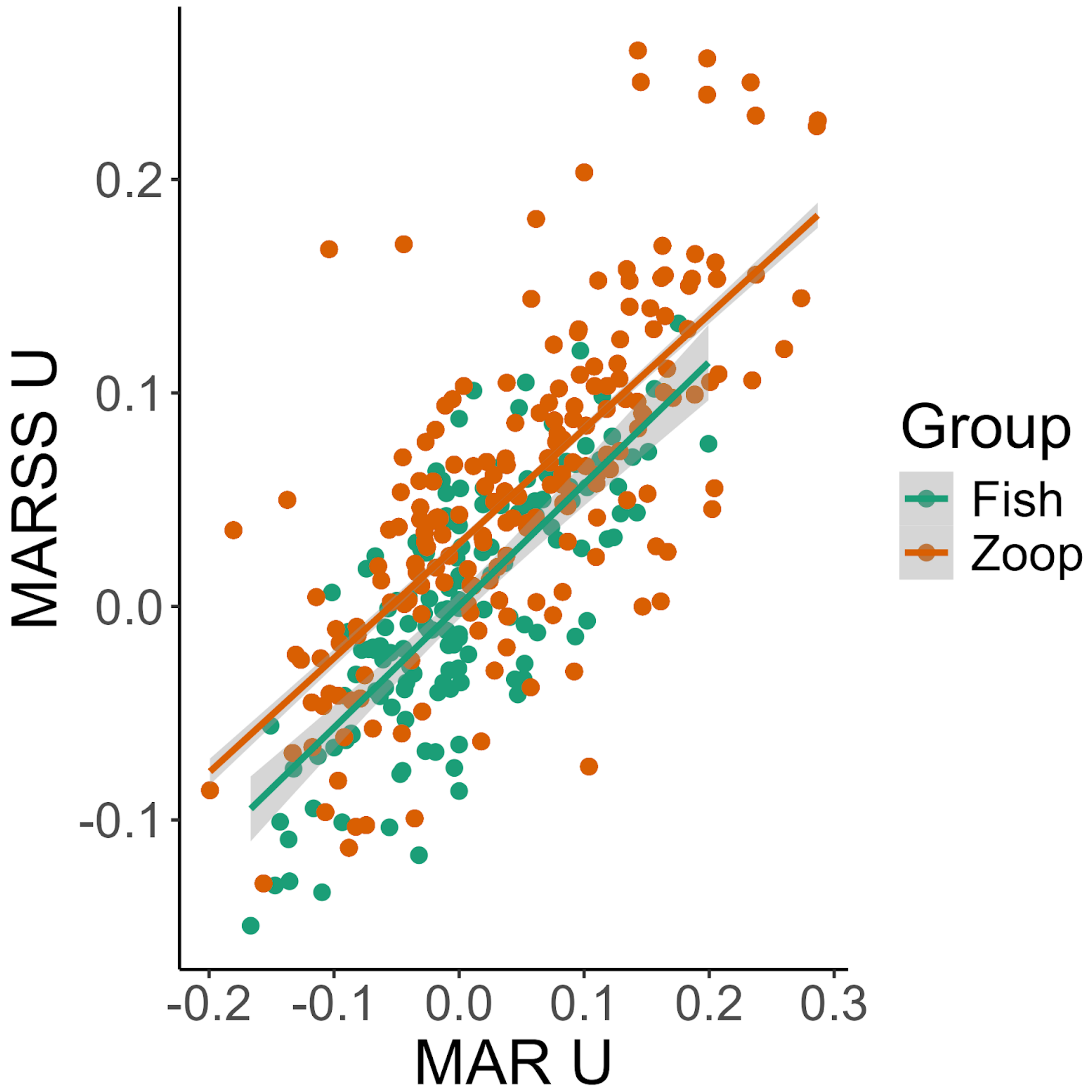

Figure S12: Comparison of MAR vs MARSS estimates for intrinsic growth rates (U). The slopes for fishes and zooplankton are both statistically significant (p<0.001) and highly similar–0.981 for fishes and 0.964 for zooplankton.

This comparison indicates that including observation error had negligible effect on estimates of U. However, MARSS-estimated Q values were consistently degenerate. To illustrate how R absorbed nearly all temporal variation, we plotted the total variance estimated by R + Q in the MARSS models against the Q estimates from the MAR models (see Fig.S13, next page).

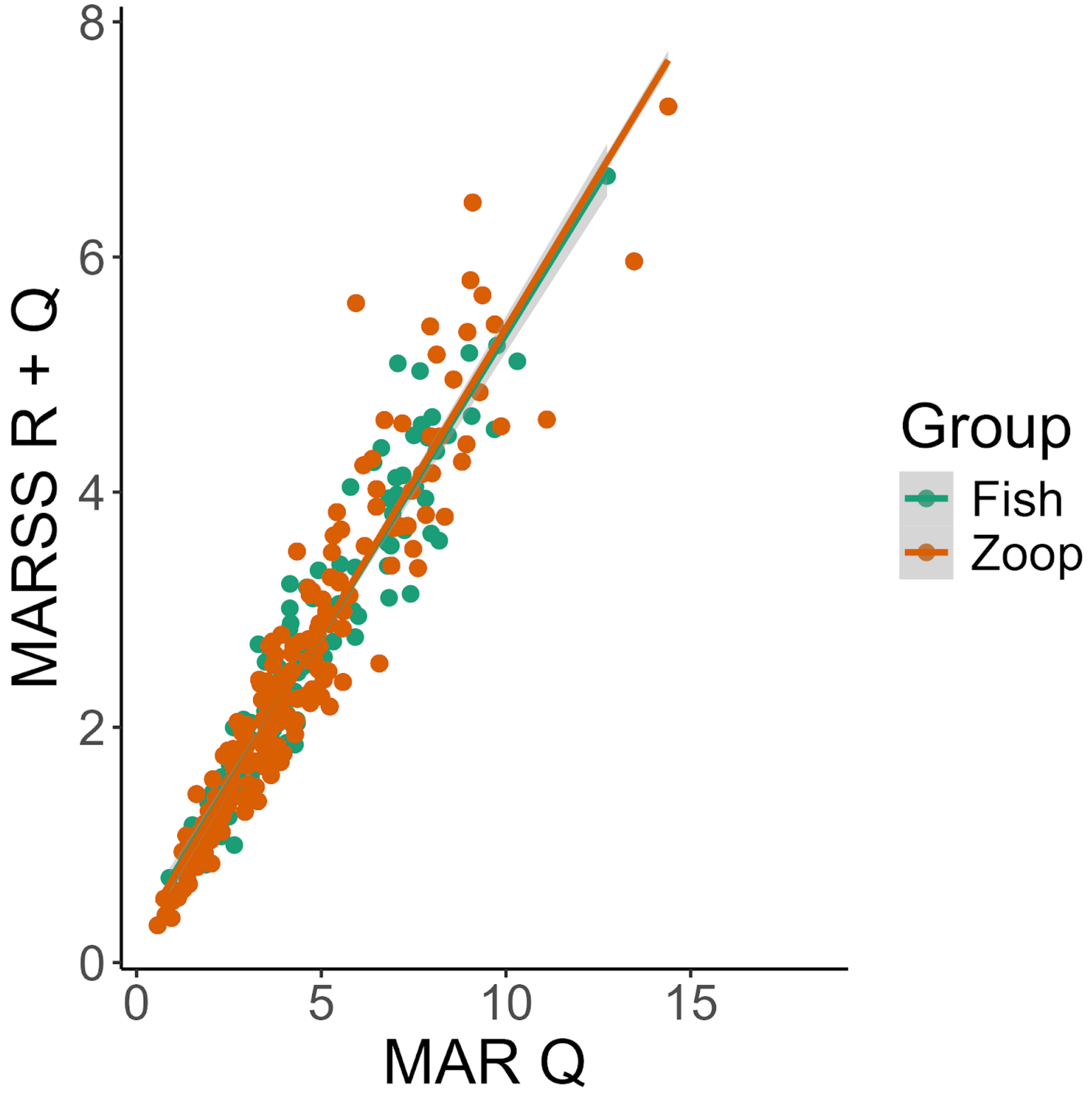

Figure S13: All variance estimates for MARSS (R + Q) and MAR (Q) models. The slopes for fishes and zooplankton are both statistically significant ($p<0.001$) and highly similar–1.79 for fishes and 1.72 for zooplankton.

Although R and Q operate on different conceptual scales, their summed variance in MARSS models closely tracks Q from MAR models, and this pattern holds when considering estimates from all taxonomic groups, months, and regions.

We note that modeling observation error is an important statistical consideration. However, given the widespread degeneracy and convergence failures we observed, we opted to use MAR

models across all species to ensure consistency in model structure and comparability of estimated trends. We acknowledge that this likely results in upwardly biased estimates of Q, which could propagate into elevated estimates of risk. Accordingly, we have framed our findings with caution, emphasizing comparative patterns, such as predator-prey divergence, over absolute magnitudes of decline risk.

Section S3: Propagation of uncertainty

To assess how uncertainty in parameter estimates for U and Q might propagate into estimates of risk and subsequent analyses, we examined multiple risk scenarios. We assessed 'best-case' scenario risk using the upper end of the bootstrapped confidence interval for U and the lower end of the confidence interval for Q (i.e., high growth rate and low process error variance), and 'worst-case' scenario risk using the lower end of the confidence interval for U and the upper end for Q (i.e., low or negative intrinsic growth and high process error variance).

Across all worst-case scenarios, we find broad patterns of divergence at the community scale with risk differing by group ($F_{1,3.974}$, p=0.046), region ($F_{1,1201.149}$, p<0.001), predator identity ($F_{1,3.974}$, p=0.046), the interactions of region by time ($F_{3,4.219}$, p=0.005), group by region ($F_{3,4.121}$, p=0.006), and the triple interaction between group, region, and time ($F_{3,2.95}$, p=0.03). For individual predators, we find significant divergences between predator and prey for Threadfin Shad ($F_{1,4.006}$, p=0.047) and Jack Silverside ($F_{1,13.940}$, p<0.001), as well as regional differences for Northern Anchovy ($F_{3,4.607}$, p=0.034) and American Shad ($F_{3,6.032}$, p<0.001) food webs, as well as the interaction between group and region ($F_{3,2.921}$, p=0.035), and region and time ($F_{3,4.965}$, p=0.002) for American Shad.

In best-case scenarios, we find similar divergence between groups ($F_{1,48.553}$, p<0.001), region ($F_{3,153.205}$ p<0.001), predator identity ($F_{6,7.347}$, p<0.001), as well as the group by region interaction ($F_{3,62.905}$, p<0.001), and region by time interaction ($F_{3,3.243}$, p=0.021). For individual predators, we find divergence between groups for Northern Anchovy ($F_{1,49.267}$, p<0.001), Pacific Herring ($F_{1,208.113}$, p<0.001), Longfin Smelt ($F_{1,12.690}$, p<0.001), American Shad ($F_{1,36.11}$, p<0.001), Striped Bass ($F_{1,32.477}$, p<0.001), and Jack Silverside ($F_{1,16.524}$, p<0.001). Similarly, we saw regional differences for Northern Anchovy ($F_{1,50.540}$, p<0.001), Longfin Smelt, ($F_{1,41.717}$, p<0.001), American Shad ($F_{1,61.595}$, p<0.001), and Threadfin Shad ($F_{1,13.989}$, p<0.001). We saw significant group by region interactions for Northern Anchovy ($F_{1,20.505}$, p<0.001), Longfin Smelt ($F_{1,20.572}$, p<0.001), American Shad ($F_{1,12.871}$, p<0.001), Striped Bass ($F_{1,27.563}$, p<0.001), and Threadfin Shad ($F_{1,7.865}$, p<0.001). Finally, we saw an interaction between group and time for Pacific Herring ($F_{1,4.781}$, p=0.035), and region and time for Longfin Smelt ($F_{1,5.251}$, p=0.023).

Taken together, parameter estimates at the bounds of our confidence intervals produce patterns broadly consistent with those generated by our maximum likelihood estimates. However, we observed substantially greater divergence between predator and prey assemblages under "best-case" scenarios compared to "worst-case" scenarios. This pattern is largely driven by risk estimates approaching asymptotic convergence at the upper end of the probability space under worst-case conditions.
